# Effects of Compound Fertilizer and Branch Fertilizer on Population Construction and Yield of Machine-Transplanted Rice

**DOI:** 10.3390/plants13172436

**Published:** 2024-08-31

**Authors:** Peng Ma, Xuehuan Liao, Keyuan Zhang, Lise Aer, Jun Deng, Erluo Yang, Rongping Zhang

**Affiliations:** School of Life Science and Engineering, Southwest University of Science and Technology, Mianyang 621010, China; mapeng7815640@163.com (P.M.); lxh000818@163.com (X.L.); ky010302@163.com (K.Z.); rm88886666@163.com (L.A.); 15528510375@163.com (J.D.); 18882457656@163.com (E.Y.)

**Keywords:** *h* *ybrid rice*, medium trace elements, tillering, chlorophyll content, yield

## Abstract

In order to study the effects of combined application of compound fertilizer and branch fertilizer on the growth and yield of machine-transplanted rice, four hybrid rice varieties were used as experimental materials, and four fertilization treatments were set up by completely random design: compound fertilizer (T0), compound fertilizer + conventional branch fertilizer (T1), compound fertilizer + (branch fertilizer − 20%) (T2), compound fertilizer + (branch fertilizer + 20%) (T3). The results showed that the branch fertilizer could effectively promote the early growth and rapid development of tillers, and increase the agronomic traits such as chlorophyll content, LAI and dry matter accumulation. Among the four varieties, the yield of the V4 variety was the highest under T3 treatment, which was 11,471.15 kg·hm^−2^, which was 37.34% higher than that of the control, and the yield increase effect was the most significant. The correlations showed that dry matter accumulation and LAI were significantly or highly significantly positively correlated with the number of effective spikes and yield, and the number of effective spikes was highly significantly positively correlated with the yield. In general, the application of pitchfork fertiliser increased the effective number of spikes and the number of grains per spike of each variety to different degrees, which effectively promoted the improvement of the rice yield.

## 1. Introduction

In recent years, the restructuring of the industrial sector and relocation of a skilled labour force from rural areas have highlighted the need to accelerate the development of mechanized crop production. The mechanization of rice planting represents a significant challenge in the comprehensive mechanization of crop production in China [1]. Modifications to the seedling cultivation process have a significant impact on the quality of rice seedlings, tillering characteristics, and the requisite levels of fertilizer and water [2]. Compared to the conventional manual transplanting method, the squat seedling stage of mechanical transplanting was observed to be relatively longer, the returning green stage of living trees was delayed by 2–3 days, and no growth was observed within 7–10 days after planting [3]. Furthermore, the frequent droughts in the early summer in the hilly rice-producing regions of northeast Sichuan, coupled with the late planting of rice after the removal of oilseed rape, wheat, and other economic crops, have led to the extension of the seedling age, prolongation of the seedling regreening period following mechanical transplanting, and weak vitality and stress resistance of the seedlings. These factors collectively affect the formation of rice yields [4]. Fertiliser application is the most important way to increase rice yields, but over-application of chemical fertilisers has led to soil pollution, ecosystem imbalance and other problems [5]. At present, organic fertiliser partially replaces chemical fertiliser as an important measure for agricultural weight loss and efficiency and has a positive role in improving the physicochemical properties of farmland soil, increasing crop productivity and reducing environmental pollution [6]. In recent years, it has been found that the rational application of organic fertilisers can increase the number and activity of soil microorganisms, increase the content of soil organic matter, and thus increase rice yield [7]. Organic–inorganic fertiliser blending improves rice yield and nitrogen use efficiency [8].

It has been demonstrated that extended seedling age in machine-transplanted rice is detrimental to the quality of the transplanting process. This was evidenced by an increase in seedling height, the emergence of yellow leaves, and a decline in rooting ability. These factors not only impede the enhancement of machine-transplanted transplanting quality but also impede the regreening of seedlings and affect tillering. This ultimately results in late tillering in the field and a reduction in the number of effective-tillering nodes. This hinders the formation of suitable and effective panicles, which are essential for achieving high yields. Consequently, this led to a reduction in rice yield [9]. Nitrogen application is one of the most direct and effective methods for increasing rice yields [10]. However, rice cultivation in China is beset with numerous challenges, including the excessive application of nitrogen fertilizers, the use of inappropriate application modes, and significant nutrient loss [11]. Accordingly, to adapt to the machine insertion mode, the fertilization method must be tailored to align it with its inherent characteristics. The utilization of varying fertilization ratios is essential to ensure the provision of the requisite nutrients for optimal crop growth and development. Kecha fertilizer is a rich source of various essential media and trace elements crucial for rice tillering. As tillering and conventional compound fertilizers, it can provide nutrition for crop growth and development, facilitate the coordination of nitrogen, phosphorus, and potassium nutrient absorption, and enhance the efficiency of fertilizer utilization [12].

When applied at an appropriate dosage, the return of green fertilizer can facilitate the early growth and rapid development of roots and tillers, enhance early growth, rapidly reflect the colour of rice after application, and promote the development of dark green leaves. This has a positive effect on the yield [13]. The application of silicon nitrogen fertilizer resulted in a notable increase in the number of panicles of rice, accompanied by a significant increase in the biomass, 1000-grain weight, and total nitrogen, phosphorus, potassium, and silicon contents of rice when compared to those observed in the nitrogen fertilizer treatment [14]. Furthermore, the application of Zn can facilitate the early growth and rapid development of rice tillers, enhance the rate of rice regreening, augment the number of effective panicles, grain number per panicle, and 1000-grain weight of rice, and increase rice yield [15,16]. The application of robust field-seeding agents has been demonstrated to facilitate the emergence of effective tillers, augment the number of effective panicles, and enhance the yield of machine-transplanted rice [17,18]. The application of new fertilizers resulted in an extension of the peak tillering period of the rice plants, accompanied by a notable increase in the number of tillers and effective panicles [19]. The application of medium- and trace-element fertilizers has been demonstrated to increase LAI and dry matter accumulation, enhancing rice yield [20].

Balanced fertilization is a critical strategy for ensuring the quality, yield, and efficiency of rice. An appropriate fertilization ratio is crucial for maintaining the soil nutrient balance, enhancing rice yield, and improving rice quality [21]. In the context of rice production, most studies have concentrated on nitrogen, phosphorus, potassium, or compound fertilizer use, which has led to a multitude of environmental concerns. There is a paucity of literature examining the impact of the combined application of compound and branch fertilizers on rice population construction and yield. It remains unclear whether branch fertilizers can be used to achieve a balance of nutrients and enhance the quality of the rice population and yield. Accordingly, this study was conducted to investigate the potential of fertilizers combined with trace elements in compound fertilizers. To enhance the survival and rooting of seedlings following mechanical transplanting, seedling regrowth, early growth, and rapid development of tillers, the effect of branch fertilizer on the population structure and yield of mechanically transplanted rice was investigated, and the relationship between the yield of mechanically transplanted rice and its growth, development, and physiological characteristics was analysed. The implementation of mechanical transplanting enhanced the quality of seedlings, facilitating the attainment of substantial rice yield. This outcome provides a theoretical foundation for the promotion of branch fertilization.

## 2. Results

### 2.1. Effects of Branch Fertilizer on Tiller Dynamics of Rice Population

**Figure 1:**
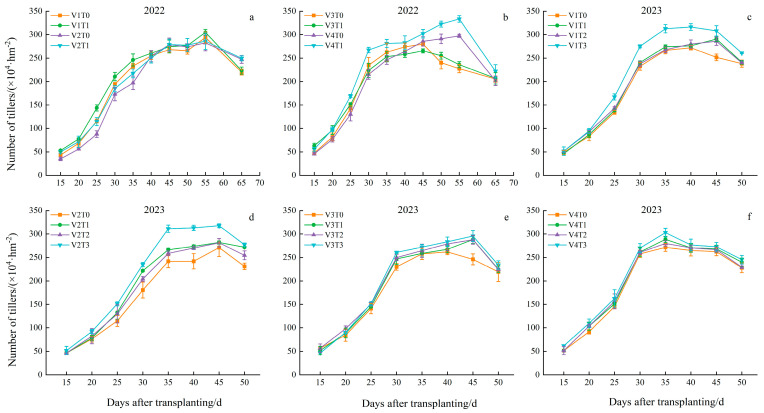
Effects of branch fertilizer on the tiller dynamics of the rice population. T0: compound fertilizer, T1: conventional branch fertilizer, T2: branch fertilizer − 20%, T3: branch fertilizer + 20%, V1: Longliangyou 534, V2: Yunliangyou 332, V3: Taifengyou 208, and V4: Nei 6 You 6368. Error bars state SD. (**a**) Number of tillers in V1 and V2 in 2022; (**b**) Number of tillers in V3 and V4 in 2022; (**c**) Number of tillers in V1 in 2023; (**d**) Number of tillers in V2 in 2023; (**e**) Number of tillers in V3 in 2023; (**f**) Number of tillers in V4 in 2023.

Analysis of tiller dynamics under the 2-year fertilization treatment revealed that with the progression of the growth process, the number of tillers of the four varieties under each treatment initially increased and subsequently decreased (Figure 1). In 2022, the highest tiller number of Nei 6 You 6368, was substantially higher than the other three varieties, with tiller numbers of 5.38%, 10.98%, and 15.68%, respectively. This demonstrates a strong tillering ability. The number of tillers reached a maximum at 55 d, except for Taifengyou, which exhibited a maximum of 208 tillers. The growth rate of “Yunliangyou 332” was the fastest between 25 and 30 d, reaching 79%. Fertilization resulted in a notable increase in the maximum number of tillers observed in the rice plants. Compared to the T0 treatment, the maximum tiller number of the four varieties under the T1 treatment increased by 3.69%, 1.42%, 0.69%, and 12.17%, respectively.

In 2023, the highest tiller number for Longliangyou 534 was significantly higher than that for the other three varieties (1.10%, 2.76%, and 1.91%, respectively). The number of tillers reached a maximum at 45 days, except for Nei 6 You 6368. The highest growth rate was observed for Nei 6 You 6368, reaching 72.70% at 25–30 days. Compared to the T0 treatment, the number of tillers observed under the different fertilization treatments was significantly higher. The number of tillers for the four varieties under the T3 treatment reached a maximum, exhibiting increases of 22.43%, 17.24%, 20.28%, and 11.54%, respectively, compared to the control. 

Significant differences were observed in the number of tillers between the different varieties over the two years and in the number of tillers under the fertilization treatment. The interaction between variety and fertilization also had a significant effect on the number of rice tillers, indicating that the application of branch fertilizer could enhance the tillering capacity of rice, increase the number of groups, and promote a higher yield.

### 2.2. Effects of Branch Fertilizer on Chlorophyll Content at the Heading Stage

**Figure 2:**
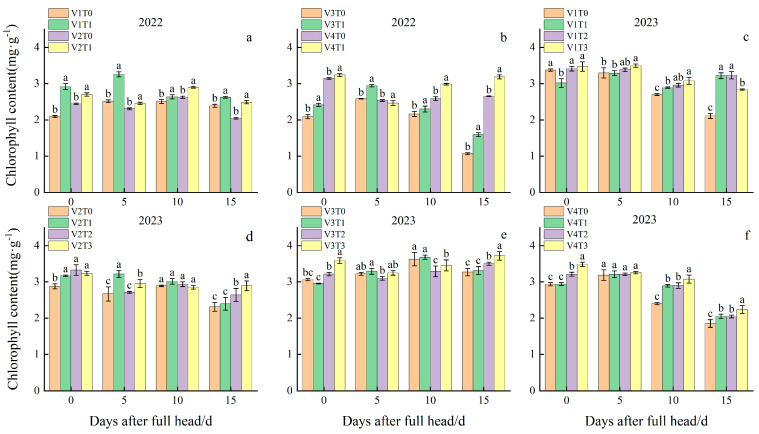
Effects of branch fertilizer on chlorophyll content at the heading stage. T0: compound fertilizer, T1: conventional branch fertilizer, T2: branch fertilizer − 20%, T3: branch fertilizer + 20%, V1: Longliangyou 534, V2: Yunliangyou 332, V3: Taifengyou 208, and V4: Nei 6 You 6368. Error bars state SD. Different letters are significantly different according to LSD (0.05). (**a**) Chlorophyll content in V1 and V2 in 2022; (**b**) Chlorophyll content in V3 and V4 in 2022; (**c**) Chlorophyll content in V1 in 2023; (**d**) Chlorophyll content in V2 in 2023; (**e**) Chlorophyll content in V3 in 2023; (**f**) Chlorophyll content in V4 in 2023.

The chlorophyll content serves as the primary index for measuring the photosynthetic capacity of plants. A higher chlorophyll content indicates greater photosynthetic capacity. The determination of chlorophyll content in rice plants at the full heading stage revealed significant differences in chlorophyll content among different varieties, fertilization treatments, and the interactions between varieties and fertilization treatments (Figure 2). At the full heading stage in 2022, the chlorophyll content of the V4 variety was 8.83%, 14.17%, and 32.87% higher than the other three varieties, respectively. The chlorophyll content of T1 at the full heading stage was 14.14% higher than T0, and the chlorophyll content of the different varieties under the fertilization treatment was T1 > T0. The chlorophyll content under the V1T1 treatment was the highest at 5 d after the full heading stage, representing a 29.42% increase compared to the control treatment V1T0. 

In 2023, the chlorophyll content of the V3 variety at the full heading stage was 7.59%, 16.16%, and 19.29% higher than the other three varieties. The chlorophyll content of T3 at the full heading stage was observed to be 11.14% higher than T0, 4.82% higher than T1, and 3.69% higher than T2. The chlorophyll contents of the different varieties under the fertilization treatment were in the following order: T3 > T2 > T1 > T0. The chlorophyll content under the V3T3 treatment was the highest 15 d after the heading stage, exhibiting a 13.56% increase relative to that of the control treatment V3T0. The results demonstrated notable variations in chlorophyll content across the different varieties. Furthermore, the application of fertilization treatments was observed to effectively mitigated the senescence rate of leaves, prolonged the functional period of flag leaves, facilitated the formation of more assimilates after flowering, and stimulated an increase in yield.

### 2.3. Effects of Branch Fertilizer on the LAI of the Main Growth Period

**Figure 3:**
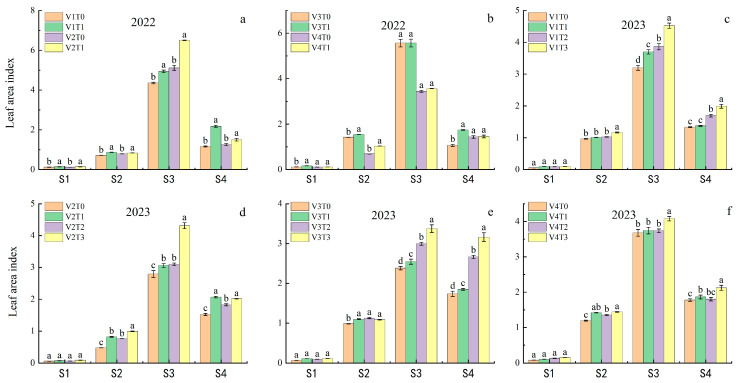
Effects of branch fertilizer on LAI of main growth period. T0: compound fertilizer, T1: conventional branch fertilizer, T2: branch fertilizer − 20%, T3: branch fertilizer + 20%, V1: Longliangyou 534, V2: Yunliangyou 332, V3: Taifengyou 208, V4: Nei 6 You 6368, S1: 15 days after transplanting, S2: 30 days after transplanting, S3: full heading stage, and S4: maturation stage. Error bars state SD. Different letters are significantly different according to LSD (0.05). (**a**) LAI for V1 and V2 in 2022; (**b**) LAI for V3 and V4 in 2022; (**c**) LAI for V1 in 2023; (**d**) LAI for V2 in 2023; (**e**) LAI for V3 in 2023; (**f**): LAI for V4 in 2023.

Analysis of the LAI over the two-year period revealed that the LAI of the four rice varieties initially increased and then decreased under the fertilization treatment (Figure 3). Significant differences in LAI were observed among the rice varieties, fertilization treatments, and the interactions between varieties and fertilization treatments at 30 days, the full heading stage, and the mature stage. At the full heading stage in 2022, the LAI of V2 was 24.99%, 4.33%, and 65.82% higher than those of the other three varieties. The highest LAI was observed for the different varieties at the full heading stage, with the LAI under the fertilization treatments showing a clear superiority of T1 over T0. The highest LAI under the V2T1 treatment was 6.50, which was 27.21% higher than the control V2T0. The LAI in the V1T1 treatment at the mature stage was the highest at 2.18, which was 87.51% higher than the control V1T0. 

In 2023, the LAI of V1 was 15.23%, 35.32%, and 0.33% higher than the other three varieties. The mature LAI of V3 was 47.27%, 26.76%, and 24.41% higher than those of the other three varieties. The highest LAI was observed at the full heading stage, with the T3 treatment exhibiting the highest LAI, followed by T2, T1, and T0. The highest LAI observed in the V1T3 treatment was 4.53, representing a 41.48% increase compared with the control V1T0. The highest LAI under the V3T3 treatment at the mature stage was 3.17, which was 82.09% higher than the control V1T0. The analysis of fertilization treatments for different varieties revealed significant differences in the LAI among the four varieties. The application of branch fertilizer resulted in a notable increase in the LAI of the different varieties, with the highest LAI observed under the T3 treatment. This indicates that the growth advantage of rice under the fertilization treatment is primarily evident during the middle and late-growth stages. This resulted in a rapid increase in leaf area during the middle growth stage and prevented premature leaf decay during the late-growth stage.

### 2.4. Effects of Branch Fertilizer on Dry Matter Accumulation and Growth Rate in the Main Growth Period

The analysis of dry matter accumulation over a two-year period revealed a growing trend in the dry matter accumulation of the four varieties under the fertilization treatment (Table 1). In addition, significant differences were observed in dry matter accumulation and growth rate among the different varieties, fertilization treatments, and varieties × fertilization. The dry matter accumulation at 15 d, 30 d, the full heading stage, and the maturity stage following fertilization treatment, as well as the growth rate from 15 d to 30 d and from 30 d to the full heading stage, were all higher than those of the T0 treatment. This indicates that the fertilization treatment significantly promoted aboveground dry matter accumulation in rice. In 2022, the growth rate from 15 to 30 days after transplanting reached its maximum in V3, and the dry matter accumulation at 15 and 30 days after fertilization was also the largest. This was significantly increased by 68.39% and 2.76%, respectively, compared with the control. The dry matter accumulation of V2 at the full heading stage and the growth rate from 30 d to the full heading stage exhibited the greatest values and were significantly increased by 16.95% and 17.75%, respectively, compared with the control T0 treatment. The highest dry matter accumulation at the maturity stage and the greatest growth rate from the full heading stage to the maturity stage were observed in V4, which exhibited significant increases of 35.25% and 89.96%, respectively, compared with the control T0 treatment. 

In 2023, among the rice varieties, the greatest dry matter accumulation at 15 and 30 days after transplanting was observed in V4T3, exhibiting increases of 3.65–40.61% and 0.44–17.85%, respectively, compared to the other three treatments. The maximum dry matter accumulation at the full heading and maturity stages was observed in V4T1, exhibiting increases of 14.73–23.15% and 8.87–22.12%, respectively, compared to the other three treatments. The growth rate from 15 to 30 days after transplanting was the most rapid in V4T1, exhibiting a significant increase of 18.10% compared with the control treatment. The growth rate from day 30 to the full heading stage was the most rapid in V1T3, exhibiting a significant increase of 23.76% compared with the control. The growth rate from the heading stage to the maturity stage was the fastest in V2T1, exhibiting a significant increase of 86.37% compared with the control. The dry matter accumulation of rice during the main growth period was in the order T3 > T1 > T2 > T0 under the different fertilization treatments. The analysis of fertilization treatments under different varieties revealed that returning green branch fertilizer significantly enhanced the dry matter accumulation and growth rate of the varieties in question. The T3 treatment demonstrated the most pronounced effects.

### 2.5. Effects of Branch Fertilizer on Rice Yield

**Figure 4:**
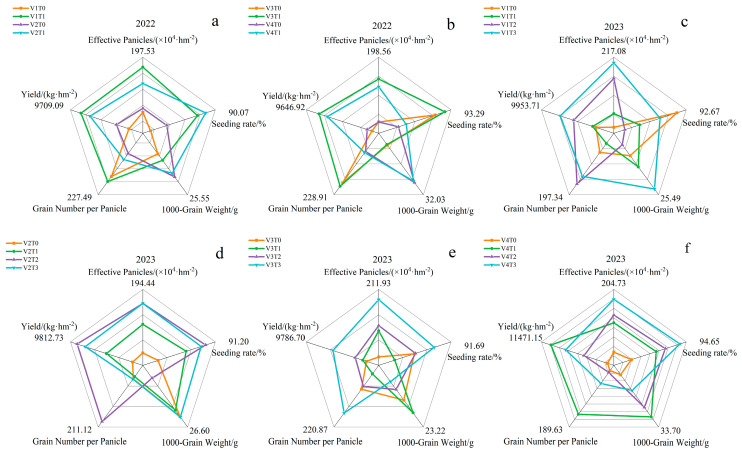
Effects of branch fertilizer on rice yield. T0: compound fertilizer, T1: conventional branch fertilizer, T2: branch fertilizer − 20%, T3: branch fertilizer + 20%, V1: Longliangyou 534, V2: Yunliangyou 332, V3: Taifengyou 208, and V4: Nei 6 You 6368. (**a**) Yield of V1 and V2 in 2022 and its components; (**b**) Yield of V3 and V4 in 2022 and its components; (**c**) Yield of V1 in 2023 and its components; (**d**) Yield of V2 in 2023 and its component factors; (**e**) Yield of V3 in 2023 and its components; (**f**) Yield and its components for V4 in 2023.

As seen in Figure 4, the effect of branch fertiliser on yield and constitutive factors of different rice varieties was significant. The application of branch fertilizer resulted in an increase in the number of effective panicles per unit area and number of grains per panicle in the four varieties, with the degree of increase varying across the four varieties. This resulted in an overall increase in the yields of the four varieties. In the 2022 harvest, V1 exhibited the highest yield at 9709.09 kg·hm^−2^. Regarding the fertilization treatment, the yield of the T1 treatment was 13.75% higher than the T0 treatment. The analysis of yield components revealed that the number of effective panicles of the four varieties under the T1 treatment increased by 6.08%, 3.30%, 12.87%, and 10.53%, respectively, compared with the control T0 treatment. In addition, the number of grains per panicle increased by 1.26%, 1.61%, 2.02%, and 0.92%, respectively, compared with the control. The final yield increased by 15.80%, 8.38%, 17.83%, and 13.21%, respectively. 

By 2023, the yield of the V4 variety was the highest, reaching 11,471.15 kg·hm^−2^. From the perspective of fertilization, the yield of the T3 treatment was 26.83% higher than the T0 treatment. This represented an increase of 11.22% compared with the T1 treatment and 9.19% compared with the T2 treatment. The analysis of yield components revealed that the seed-setting rate under the V1T3 treatment exhibited a 0.62% decline compared to the control T0 treatment. Conversely, the effective panicle number, 1000-grain weight, and grain number per panicle were demonstrated by a 18.54%, 2.62%, and 3.09% increase, respectively. Consequently, the final yield experienced a 24.55% enhancement. The 1000-grain weight in the V2T2 treatment exhibited a decrease of 1.45%, whereas the effective panicle number, seed-setting rate, and grain number per panicle increased by 11.18%, 4.52%, and 3.41%, respectively. Consequently, the final yield increase by 18.48%. The 1000-grain weight decreased by 1.05% under the V3T3 treatment, whereas the effective panicle number, seed-setting rate, and grain number per panicle increased by 27.16%, 3.11%, and 7.54%, respectively. This resulted in a final yield increase of 39.51%. The effective panicle number, seed-setting rate, 1000-grain weight, and grain number per panicle increased by 8.72%, 1.49%, 1.24%, and 22.95%, respectively, under V4T1 treatment, resulting in a final yield increase of 37.34%. These findings indicate that the increase in the effective panicle number and grain number per panicle is a primary factor contributing to the elevated rice yield following the application of branch fertilizer. The use of branch fertilizer had a pronounced impact on the enhancement of rice yield.

The analysis of variance as shown in Table 2, showed that in 2022, there were significant or highly significant differences between varieties in terms of rice yield and seed-setting rate, 1000-grain weight, and grain number per panicle. Significant differences were observed in rice yield, effective panicle number, and seed-setting rate across different fertilization treatments. In addition, significant differences were noted in yield and seed-setting rates owing to the interaction between variety and treatment. In 2023, there were highly significant differences in the rice yield, effective panicle number, seed-setting rate, 1000-grain weight, and grain number per panicle among the different varieties. Significant differences were observed in the rice yield, effective panicle number, and seed-setting rate across the fertilization treatments. Significant differences were observed in yield, seed-setting rate, and grain number per panicle under the interaction of variety and treatment.

### 2.6. Correlation Analysis of Rice Yield Components and Agronomic Traits

**Figure 5:**
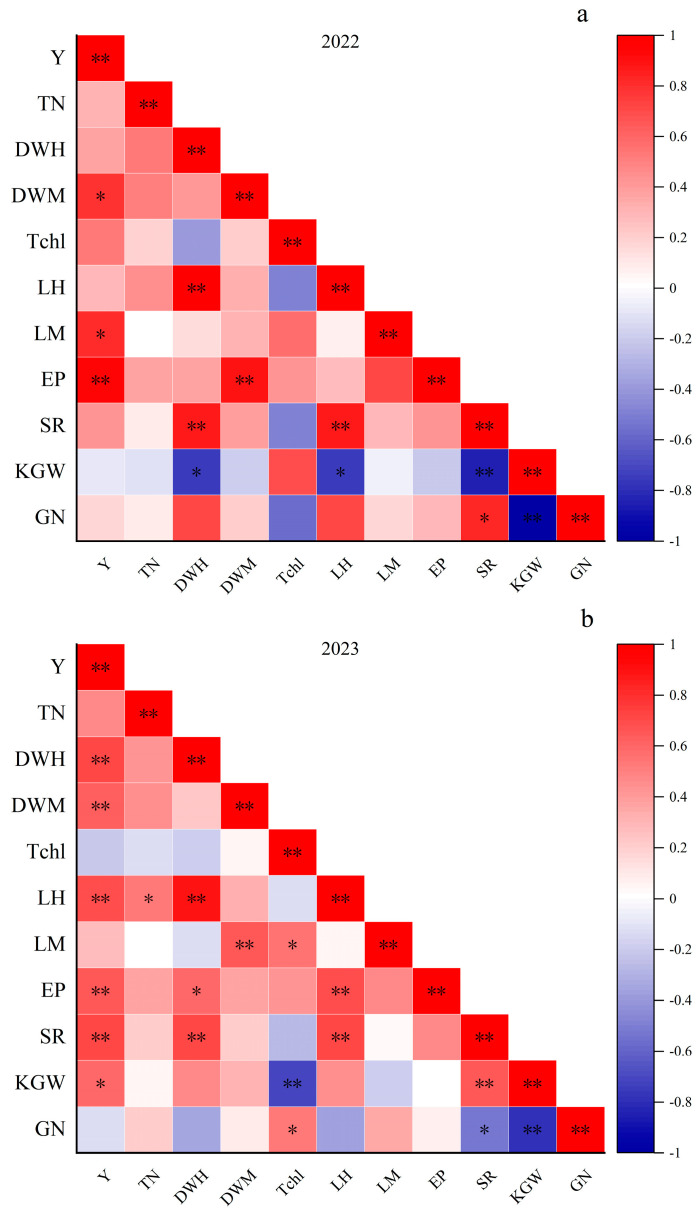
Correlation analysis of rice yield components and agronomic traits. Y: Yield, TN: tiller number, DWH: dry matter accumulation at full heading stage, DWM: dry matter accumulation in the mature stage, Tchl: total chlorophyll content, LH: leaf area index at full heading stage, LM: leaf area index at maturity, EP: effective Panicles, SR: seeding rate, KGW: 1000-Grain Weight, GN: grain number per panicle. * and ** mean significance at the 0.05 and 0.01 probability levels, respectively. (**a**) Relevance analysis for 2022; (**b**) Relevance analysis for 2023.

Correlation analysis revealed that (Figure 5a), in 2022, among the yield components of rice, EP demonstrated a significant positive correlation with Y and SR, whereas GN exhibited a significant negative correlation with KGW. In addition, GN displayed a significant positive correlation with SR. Among the agronomic traits of rice during the entire growth period, DWM and LM exhibited a significant positive correlation with Y; LH demonstrated a significant positive correlation with DWH; DWM exhibited a significant positive correlation with EP; DWH and LH exhibited a significant positive correlation with SR; and DWH and LH exhibited a significant negative correlation with KGW. 

In 2023 (Figure 5b), among the yield components of rice, EP and SR were significantly positively correlated with Y, and Y, SR, and KGW were significantly or very significantly positively correlated. Conversely, SR, KGW, and GN were significantly or very significantly negatively correlated with GN. In addition, KGW and SR were significantly and positively correlated. In accordance with the agronomic traits of rice throughout its entire growth period, DWM, DWH, and LH exhibited a significant positive correlation with Y; TN demonstrated a significant positive correlation with LH; EP, LH, and SR exhibited a significant or very significant positive correlation with DWH; DWM exhibited a significant positive correlation with LM; GN and LM exhibited a significant positive correlation with Tchl; EP and SR exhibited a significant positive correlation with LH; and KGW exhibited a significant negative correlation with Tchl.

## 3. Discussion

### 3.1. Effects of Compound Fertilizer and Branch Fertilizer on Population Construction and Dry Matter Accumulation of Rice

Previous research has demonstrated that the application of compound fertilizers can facilitate the transformation of rice matter, enhance the quantity and velocity of dry matter translocation, and augment the rice harvest index [22]. A reasonable distribution of nitrogen fertilizer can facilitate the regreening and effective tillering of rice, enhance the accumulation of dry matter in the aboveground parts, expand the photosynthetic area of leaves, and elevate the light energy utilization rate of the subsequent population [23]. The application of nitrogen fertilizer has been demonstrated to result in a corresponding increase in the biological yield of machine-transplanted rice with a prolonged seedling period. This was accompanied by an increase in the accumulation of dry matter in stems, leaves, and panicles. The application of nitrogen fertilizer at a ratio of 4:3:3 resulted in the highest accumulation of dry matter and root dry weight [24]. The application of silicon (Si) and zinc (Zn) fertilizers promotes early growth and rapid development of tillers, increases the number of tillers in rice, enhances the LAI of rice, and augments biomass accumulation [25,26,27]. The application of magnesium (Mg) and zinc (Zn) fertilizers has been shown to increase the number of effective tillers and panicles in rice, enhancing agronomic traits and markedly elevating rice yields [28]. The results demonstrated that the V4 variety exhibited the most pronounced improvement in the number of tillers, with the greatest increase observed under the T3 treatment relative to the other treatments. Following the fertilization treatment, the number of tillers in rice was observed to have been effectively improved, indicating that the regulation of branch fertilizer on rice occurred from the early stage of growth. This could promote the formation of a larger group of effective tillers, laying the foundation for high yield. 

Rice leaves are the primary site of photosynthetic production, and the accumulation of photosynthetic products is the principal determinant of rice grain yield. This process also serves as a crucial foundation for dry matter accumulation and rice yield [29]. A higher chlorophyll content in rice leaves indicates greater photosynthetic capacity, which can delay the onset of senescence in plants and ensure the transport of nutrients to the grains [30,31]. Zn has been demonstrated to enhance the chlorophyll content of rice plants, facilitate the photosynthetic chemical reaction of chlorophyll, and stimulate the synthesis and metabolism of carbohydrates in plants [32]. The findings of this study indicate that fertilization markedly enhanced the LAI and leaf chlorophyll content of rice during the primary growth phase, particularly during the mid- and late-growth stages. The LAI and chlorophyll content significantly increased by 41.48% and 29.42%, respectively, with the most pronounced effect observed in the T3 treatment. It has been demonstrated that various trace elements, including silicon, zinc, and magnesium, present in branch fertilizers, may play a role in regulating photosynthesis, respiration, and other plant metabolic processes. This can lead to an increase in rice leaf material production capacity and accumulation in the later stages of growth, improved nutrient supply, and fulfilment of grain-filling material requirements. Ultimately, this can cause an enhancement of crop yield, a finding that aligns with previous research. 

As a vital organ for photosynthesis, an expansion in the leaf area allows plants to use light, facilitating the production of photosynthetic products. This enhances the growth rate and dry matter accumulation in crops. The dry matter weight of the aboveground parts indicates the state of dry matter accumulation in plants, which provides the energy necessary for the transport of dry matter to grains, thus ensuring a high rice yield [33,34]. In the context of rice yield formation, dry matter accumulation represents a basis for yield formation, and enhancing the dry matter yield of rice is a pivotal strategy for achieving high rice yields [35,36]. A significant positive correlation was observed between the rice yield and dry matter accumulation from heading to maturity [37]. The results demonstrated that dry matter accumulation under the fertilization treatment was higher during the main growth period of rice, with a more pronounced difference between treatments in the latter stages of growth. Dry matter accumulation in each treatment was T3 > T1 > T2 > T0. Furthermore, dry matter accumulation at the full heading stage and the LAI at the full heading stage exhibited a significant or extremely significant positive correlation with the number of effective panicles and seed-setting rate. Dry matter accumulation at the full heading stage, dry matter accumulation at the mature stage, and leaf area index at the full heading stage were significantly positively correlated with yield, which is consistent with the findings of previous studies. The results demonstrated that the application of branch fertilizer led to an increase in the number of stems and tillers of rice, enhanced dry matter accumulation, guaranteed the transportation and accumulation of substances to grains at the filling stage, increased effective panicles at the maturity stage, improved the panicle rate of rice, and established a foundation for the development of high-yield populations.

### 3.2. Effects of Compound Fertilizer and Branch Fertilizer on Yield Components and Rice Yield

The yield of rice is contingent upon several factors, including the number of effective panicles per unit area, number of grains per panicle, weight of 1000 grains, and seed-setting rate. The number of effective panicles and number of grains per panicle serve as pivotal indicators for enhancing rice yield [38]. The application of fertilizers is a crucial strategy for regulating crop yields in agricultural production. Besides nitrogen, phosphorus, and K, other essential elements, including Zn, Mg, and Si, are indispensable for optimal crop growth [39]. Prior research has demonstrated that the utilization of compound fertilizer can enhance the number of panicles per unit area and the number of grains per panicle, consequently elevating rice yield [22]. An appropriate fertilization ratio will cause an increase in panicle number, grain number per panicle, and 1000-grain weight of double-season mechanized rice, which will lead to an increase in yield [40]. A ratio of 7:3 between basal-tillering and panicle fertilizers resulted in a notable increase in dry matter accumulation and rice yield [41]. 

The application of Si and Zn fertilizers, besides conventional fertilization, has been demonstrated to enhance rice yield and yield components [42]. The application of Zn and Mg fertilizers has effectively shortened the regreening time of seedlings, promoted the occurrence of rice tillering, increased the effective panicle number, and increased the rice yield by 7.6% [43,44]. The application of Si, Zn, boron, and other medium and trace-element fertilizers has been demonstrated to increase the number of grains per panicle, 1000-grain weight, and the seed-setting rate of rice [45,46]. The results demonstrated that the formula fertilization treatment had a significant effect on the growth and yield components of rice. Grain yield exhibited a significant or extremely significant positive correlation with the number of effective panicles, number of grains per panicle, and 1000-grain weight [47]. The findings revealed that, compared to the control, the average yield of the four varieties increased by 20.18%, 13.43%, 28.67%, and 25.36% under the two-year fertilization treatment. The highest yield was observed in the V4 variety, with an average of 11,471.15 kg·hm^−2^. From the perspective of fertilization treatment, the yield of the T3 treatment was 26.83% higher than the control T0 treatment, indicating that the application of different gradients of reviving branch fertilizer had disparate effects on the yield and composition factors of rice. Among these effects, the application of T3 was the most pronounced. A significant positive correlation was observed between the number of effective panicles and yield among the rice yield components under the two-year fertilization treatment. The application of branch fertilizer resulted in a notable increase in the number of effective panicles and grains per panicle in rice plants. In addition, the seed-setting rate and 1000-grain weight increased, enhancing rice yield. The primary reason for these outcomes may be the regulation of diverse trace elements, including silicon, zinc, and magnesium, in the fertilizer during the vegetative growth and filling process of rice. The precise mechanism by which this occurs requires further investigation.

## 4. Materials and Methods

### 4.1. Test Site and Materials

The experiment was carried out in the experimental field of Southwest University of Science and Technology (31°53′ N,104°70′ E) from 2022 to 2023. The soil fertility of the test was above average, and the farmland was closed in winter. The basic physical and chemical properties of topsoil (0~20 cm) are as follows: total nitrogen 1.65 g/kg, total phosphorus 0.73 g/kg, total potassium 15.49 g/kg, alkali-hydrolyzable nitrogen 195.38 g/kg, available phosphorus 18.64 g/kg, available potassium 130.18 g/kg, pH value 7.10. These varieties were Longliangyou 534, Yunliangyou 332, Taifengyou 208, and Nei 6 You 6368. In this experiment, the conventional compound fertilizer is Jinzhengda 3 + 3 compound fertilizer (N-P_2_O_5_-K_2_O is 18-12-10) produced by Jinzhengda Ecological Engineering Group Co., Ltd. (Linyi, China). The branch fertilizer + ETDA silicon (also known as rice tillering fertilizer), referred to as branch fertilizer, produced by Shandong Shitianbu Agricultural Development Co., Ltd. (Taian, China), is a medium and trace element water-soluble fertilizer, which is rich in various medium and trace elements required for rice tillering. The amount is 15 kg·hm^−^^2^.

### 4.2. Experimental Design and Field Management

The experiment was conducted in a two-factor completely randomised design, with four fertilization treatments set up for factor A: (T0) compound fertilizer, (T1) compound fertilizer + conventional branch fertilizer, (T2) compound fertilizer + (branch fertilizer − 20% concentration), and (T3) compound fertilizer + (branch fertilizer + 20% concentration), with three replications for each treatment. The B factor was based on four rice varieties as test materials, namely Longliangyou 534, Yunliangyou 332, Taifengyou 208, and Nei 6 You 6368, which are denoted by V1, V2, V3, and V4, respectively. In 2022, the seedlings were cultivated from April 11 onwards, with the transplantation of the rice crop on 24 May. In 2023, the seedlings were cultivated from April 26 onwards, with the transplantation of the rice crop on 28 May. The dimensions of the transplant were 36 cm × 18 cm. The area of each plot was 40 m^2^, and the application rate of conventional compound fertiliser (in terms of N) was discounted at 150 kg·hm^−^^2^, and the fertiliser rate was applied as base fertiliser:tiller fertiliser:spike fertiliser = 5:3:2. The reviving branch fertilizer and tillering fertilizer were combined and applied to the soil surface seven days after transplanting. Additional cultivation and management measures were implemented under local high-yield cultivation guidelines (See Table 3).

### 4.3. Determination Items and Methods

#### 4.3.1. Tillering Dynamics

In the initial tillering phase, 30 plants exhibiting uniform growth, no disease, and an equivalent number of tillers were selected to investigate tiller dynamics until the number of tillers reached a stable equilibrium.

#### 4.3.2. Dry Matter Accumulation and LAI

The number of tillers in each plot was measured during the tillering, full heading, and mature rice stages, and the average number of tillers per hole was calculated. Based on these observations, five representative plants were selected from each plot and the length and width of the leaves were measured. The leaf area index (LAI) was calculated. The aboveground parts of the five holes of rice were divided into stems, leaves, and panicles (only the stems and leaves were divided into stems and leaves at the peak tillering stage of rice), deactivated at 105 °C for 30 min, dried at 75 °C to a constant weight, and the dry matter weight of each part was measured.

#### 4.3.3. Chlorophyll Content

Chloroplast pigments were extracted using a specified method. The OD values were then measured using an ultraviolet spectrophotometer (UV-752, JINGHUA instruments Co., Ltd., Shanghai, China) at wavelengths of 645 and 663 nm. Finally, the chlorophyll a, chlorophyll b, and total chlorophyll contents were calculated.

#### 4.3.4. Yield and Its Components

At the mature stage, five plants were randomly selected from each plot, according to the average number of effective panicles. The rice panicles were then removed, placed in an envelope, and transported to the laboratory for planting. An indoor test was conducted to ascertain the number of filled grains, number of shrunken grains, 1000-grain weight, number of effective panicles, number of grains per panicle, and seed-setting rate.

#### 4.3.5. Data Analysis

Analyses of variance (ANOVA) and least significant difference (LSD) tests were used to compare data using SPSS v23 (Chinese version v22.0.0.0) (Statistical Product and Service Solutions Inc., Chicago, IL, USA), with a significance threshold of *p* < 0.05. Figures were constructed using Origin Pro 2023 (OriginLab, Northampton, MA, USA).

## 5. Conclusions

This study proposes the application of silicon, zinc and magnesium fertilisers on the basis of conventional fertiliser by addressing the urgent problems in production, and to study the effects of different fertiliser ratios on the construction of machine-plugged rice groups and yield formation. The results showed that the application of branch fertiliser + 20% in combination with conventional fertiliser can increase the number of tillers per unit area of the rice population, which can effectively regulate the growth and development process of rice, and thus increase the yield of rice. It provides a theoretical basis for high-yield and high-quality cultivation technology of machine-plugged rice and is of great significance for the application and promotion of branch fertiliser. And in this study, compared with the other four varieties, the Nei 6 You 6368 variety had the highest yield of 11,471.15 kg·hm^−2^, which was 37.34% higher than the control treatment. It indicates that this variety is suitable for high-yield cultivation with branch fertiliser in the hilly rice areas of east Sichuan.

## Figures and Tables

**Table 1:** Effects of branch fertilizer on dry matter accumulation and growth rate in the main growth period.

Year	Variety	Treatment	Dry Matter Accumulation/(kg·hm^−2^)				Crop Growth Rate/[g/(d·m^2^)]		
			S1	S2	S3	S4	S1–S2	S2–S3	S3–S4
2022	1	T0	120.06 b	889.40 b	11,429.01 b	16,558.13 b	5.13 a	25.71 b	15.09 b
		T1	147.43 a	918.21 a	13,308.64 a	16,895.58 a	5.14 a	30.22 a	10.55 a
		Mean	133.74	903.81	12,368.83	16,726.85	5.13	27.96	12.82
	2	T0	125.51 a	788.07 b	13,582.82 b	17,450.10 b	4.41 b	31.21 b	11.38 b
		T1	130.66 a	817.39 a	15,885.29 a	18,118.31 a	4.58 a	36.75 a	6.57 a
		Mean	128.09	802.73	14,734.05	17,784.21	4.50	33.98	8.97
	3	T0	99.28 b	1005.14 b	13,649.69 b	15,084.88 b	6.04 b	30.84 b	4.22 b
		T1	167.18 a	1032.92 a	13,860.08 a	18,701.13 a	5.77 a	31.29 a	14.24 a
		Mean	133.23	1019.03	13,754.89	16,893.00	5.91	31.06	9.23
	4	T0	117.80 a	776.75 b	9355.97 b	13,829.73 b	4.39 b	20.92 b	13.16b
		T1	120.88 a	960.90 a	10,202.68 a	18,704.22 a	5.60 a	22.54 a	25.00a
		Mean	119.34	868.83	9779.32	16,266.98	5.00	21.73	19.08
F Value		V	22.97 **	6513.11 **	4368.22 **	160.90 **	5752.63 **	4339.37 **	800.63 **
		T	344.55 **	3619.18 **	1619.31 **	2246.08 **	1327.13 **	1448.01 **	353.22 **
		V × T	116.54 **	1200.36 **	215.00 **	493.24 **	1776.53 **	225.56 **	737.40 **
2023	1	T0	42.90 d	935.70 d	10,166.15 d	11,801.44 d	5.95 c	22.51 d	4.81 c
		T1	59.31 b	951.13 c	11,572.53 b	12,191.36 c	5.95 c	25.90 b	1.82 d
		T2	55.15 c	981.28 b	10,751.03 c	14,116.77 b	6.17 b	23.83 c	9.90 b
		T3	64.66 a	1049.38 a	12,477.37 a	16,177.99 a	6.57 a	27.87 a	10.88 a
		Mean	55.50	979.37	11,241.77	13,571.89	6.16	25.03	6.85
	2	T0	44.29 d	727.93 d	8744.86 d	13,408.95 d	4.56 c	19.55 d	13.72 d
		T1	61.21 b	825.77 b	9158.44 c	17,851.85 b	5.10 b	20.32 c	25.57a
		T2	53.29 c	806.84 c	9533.95 b	15,242.28 c	5.02 b	21.28 b	16.79 c
		T3	63.53 a	860.08 a	11,185.70 a	18,217.08 a	5.31 a	25.19 a	20.68 b
		Mean	55.58	805.16	9655.74	16,180.04	5.00	21.59	19.19
	3	T0	55.45 d	760.29 c	8075.41 d	14,116.77 d	4.70 d	17.84 d	17.77 c
		T1	83.75 a	861.94 a	8697.02 c	14,761.83 c	5.19 b	19.11 c	17.84 c
		T2	64.40 c	866.26 a	8916.66 b	16,623.97 b	5.34 a	19.63 b	22.67 a
		T3	79.22 b	837.96 b	9735.60 a	16,884.77 a	5.06 c	21.70 a	21.03 b
		Mean	70.71	831.61	8856.17	15,596.84	5.07	19.57	19.83
	4	T0	62.98 d	965.84 c	10,195.47 d	14,974.79 d	6.02 d	22.51 d	14.06 c
		T1	67.20 c	1133.23 a	12,555.56 a	18,286.52 a	7.11 a	27.86 a	16.86 a
		T2	85.44 b	1033.43 b	10,688.78 c	15,727.88 c	6.32 c	23.55 c	14.82 b
		T3	88.56 a	1138.27 a	10,943.93 b	16,797.33 b	7.00 b	23.92 b	17.22 a
		Mean	76.05	1067.70	11,095.94	16,446.63	6.61	24.46	15.74
F Value		V	1186.81 **	3522.36 **	2439.91 **	3344.71 **	3352.91 **	2083.75 **	3929.29 **
		T	974.72 **	641.52 **	1064.89 **	4007.79 **	438.87 **	960.50 **	460.51 **
		V × T	119.69 **	71.78 **	206.10 **	829.27 **	87.52 **	209.29 **	331.07 **

T0: compound fertilizer, T1: conventional branch fertilizer, T2: branch fertilizer − 20%, T3: branch fertilizer + 20%, V1: Longliangyou 534, V2: Yunliangyou 332, V3: Taifengyou 208, V4: Nei 6 You 6368, S1: 15 days after transplanting, S2: 30 days after transplanting, S3: full heading stage, and S4: maturation stage. Different letters are significantly different according to LSD (0.05). V, T and V × T represent the interaction of variety, treatment, variety and treatment, respectively. ** mean significance at the 0.01 probability levels.

**Table 2:** Variance analysis of branch fertilizer on rice yield and yield component factors.

Year	Treatment	Effective Panicles/ (×10^4^·hm^−2^)	Seeding Rate/%	1000-Grain Weight/g	Grain Number Per Panicle	Yield/(kg·hm^−2^)
2022	V	3.18	2609.38 **	863.11 **	97.92 **	11.54 *
	T	75.52 **	1547.32 **	0.31	2.43	1377.08 **
	V × T	4.736	39.25 **	2.28	0.09	26.22 **
2023	V	13.02 **	1074.92 **	1137.38 **	103.43 **	578.10 **
	T	79.38 **	277.98 **	0.93	3.3	610.33 **
	V × T	3.31	127.37 **	0.74	8.92 **	119.93 **

Note: V, T and V × T represent the interaction of variety, treatment, variety and treatment, respectively. * and ** mean significance at the 0.05 and 0.01 probability levels, respectively.

**Table 3:** The amount of compound fertilizer and branch fertilizer under different treatments.

Treatment	Compound Fertilizer/kg	Branch Fertilizer/g
(T0) compound fertilizer	3.33	0
(T1) compound fertilizer + conventional branch fertilizer	3.33	60
(T2) compound fertilizer + (branch fertilizer − 20% concentration)	3.33	48
(T3) compound fertilizer + (branch fertilizer + 20% concentration)	3.33	72

## Data Availability

The data presented in this study are available upon request from the authors.
